# Shade treatment affects structure and recovery of invasive C4 African grass *Echinochloa pyramidalis*

**DOI:** 10.1002/ece3.1434

**Published:** 2015-03-01

**Authors:** Hugo López Rosas, Patricia Moreno-Casasola, Verónica E Espejel González

**Affiliations:** 1 Estación El Carmen, Instituto de Ciencias del Mar y Limnología, UNAMCiudad del Carmen, Campeche, Mexico; 2 Red de Ecología Funcional, Instituto de Ecología, A.C.Xalapa, Veracruz, Mexico

**Keywords:** Biological invasion, cattle grazing, *Echinochloa pyramidalis*, Gulf of Mexico, La Mancha, Mexico, non-native grasses, *Pontederia sagittata*, removal experiment, *Sagittaria lancifolia*, Veracruz

## Abstract

*Echinochloa pyramidalis* (Lam.) Hitchc. & Chase is an African grass with C4 photosynthesis, high biomass production, and high vegetative propagation that is tolerant to grazing and able to grow in flooded and dry conditions. Thus, it is highly invasive in tropical freshwater marshes where it is intentionally planted by ranchers to increase cattle production. This invasion is reducing plant biodiversity by increasing the invader's aerial coverage, changing wetland hydrology and causing soil physicochemical changes such as vertical accretion. Reducing the dominance of this species and increasing the density of native wetland species is a difficult, expensive, and time-consuming process. We applied a series of disturbance treatments aimed at eliminating *E. pyramidalis* and recovering the native vegetation of a partially invaded freshwater marsh. Treatments included physical (cutting, soil disking, transplanting individuals of the key native species *Sagittaria lancifolia* subsp. *media* (Micheli) Bogin, and/or reducing light with shade mesh) and/or chemical (spraying Round-Up™ herbicide) disturbances. At the end of the experiment, four of the five treatments used were effective in increasing the cover and biomass of native species and reducing that of *E. pyramidalis*. The combination of these treatments should be used to generate a proposal for the restoration of tropical wetlands invaded by non-native grasses. A promising treatment is using soil disked to soften the soil and destroy belowground structures such as roots and rhizomes. This treatment would be more promising if combined with the use of shade cloth. If it is desirable not to impact the soil or if there is not enough budget to make an effort to include active restoration disking soil, the use of shade cloth will suffice, although the recovery of native vegetation will be slower.

## Introduction

Biological invasion is a subject of concern worldwide. Compared to prehistoric invasions, anthropogenic, modern biological invasions are the cause of a global change without precedent (Ricciardi 2007). Invasions by grasses are harmful to natural ecosystems and are related to human activities, including that of raising cattle. Grass invasions can affect a wide variety of the attributes, functions, and processes of natural ecosystems such as biodiversity, productivity, biogeochemical cycles, food webs, disturbance patterns, and hydrological cycles, among others (Williams and Baruch 2000; D'Antonio et al. 2011). In South America alone it is estimated that 53 million hectares of rainforest in the Amazon Basin of Brazil have been converted to grassland, along with some 40 million hectares of the tropical savanna native to Colombia, Venezuela, and Brazil (Matthews and Brand 2005).

Tropical freshwater swamps and marshes have also been affected by this type of invasion after the introduction of flood tolerant African grasses. Under wet and flood conditions, non-native flood tolerant African grasses may have advantages over native species because they are away from their native herbivores, they exhibits characteristics of successful invasive species, such as rapid growth, high biomass, vegetative propagation, and can withstand the limitation of low nutrient concentrations (Mack et al. 2000). In large areas of wetlands of southeast Mexico (including the states of Veracruz, Tabasco and Campeche), *Echinochloa pyramidalis* (Lam.) Hitchc. & Chase, our focal African grass species, is becoming a problematic invader (Skerman and Riveros 1990; López Rosas et al. 2005; López Rosas and Moreno-Casasola 2012). This species is used as forage for livestock in tropical American coastal marshes. Ranchers prefer it because it “dries the area and builds soil” (Melgarejo-Vivanco 1980), and because of its high productivity (Acioli de Abreu et al. 2006; Andrade et al. 2008; Braga et al. 2008). In the long term, it can drastically alter the functions and biodiversity of wetlands (López Rosas et al. 2005). The cost of reversing this may be untenable. In Guyana alone, the federal government has spent over U.S. $ 3.4M to control this invasive species (Ministry of Agriculture of Guyana 2008; EPA Guyana 2011).

To develop effective proposals for the ecological restoration of ecosystems invaded by African grasses, it is first necessary to understand the biology of these species, to identify the features that make them particularly invasive in a given environment, and to take particular note of those features absent from the native vegetation. *Echinochloa pyramidalis* has common features with the most invasive African grasses. This is a tall (max. height: 2 m) plant with tolerance to foraging, intense vegetative propagation, high production of above and belowground organic matter, C4 photosynthesis (Williams and Baruch 2000; Baruch and Jackson 2005; Osborne and Freckleton 2009), and a wide tolerance to drought and flooding (Da Silva et al. 2001; López Rosas et al. 2005; Acioli de Abreu et al. 2006; López Rosas and Moreno-Casasola 2012).

This study was conducted in a freshwater marsh that had been partially invaded by *E. pyramidalis*. In a previous experiment, López Rosas et al. (2006) evaluated the effects of mechanical (cutting or soil disking) and chemical (spraying Round-Up™ herbicide) disturbance treatments on the plant community of this freshwater marsh, predicting that intense disturbance would eliminate this African grass from the experimental plots. Over a 9-month period, they analyzed species cover, richness, and diversity in experimental plots and found that the treatment that best reduced the dominance of *E. pyramidalis* and increased the diversity of native species was soil disking, because it led to the germination of the seed bank of many native species. Nevertheless, these species were not competitive enough to prevent the reinvasion by *E. pyramidalis*. Even when spraying herbicide on the invader, after 9 months, *E. pyramidalis* recovered in all the treatments and again became the dominant species. These authors suggested that the successful invasion of *E. pyramidalis* and the difficulty in eliminating it from the experimental plots was a result of the life form characteristics of this species (tolerance to grazing, large biomass production, C4 photosynthesis, and general life history characteristics of Poaceae). Many of these characteristics were neither present nor dominant in the indigenous marsh species. To test this idea, we designed a new experiment with more aggressive treatments against invasive species taking into account the needs of a C4 species and delaying its recovery to favor native species. We applied a series of physical and chemical disturbance treatments aimed at eliminating the invading grass and helping recovery of native marsh species.

## Materials and Methods

### Study site

**Figure 1:**
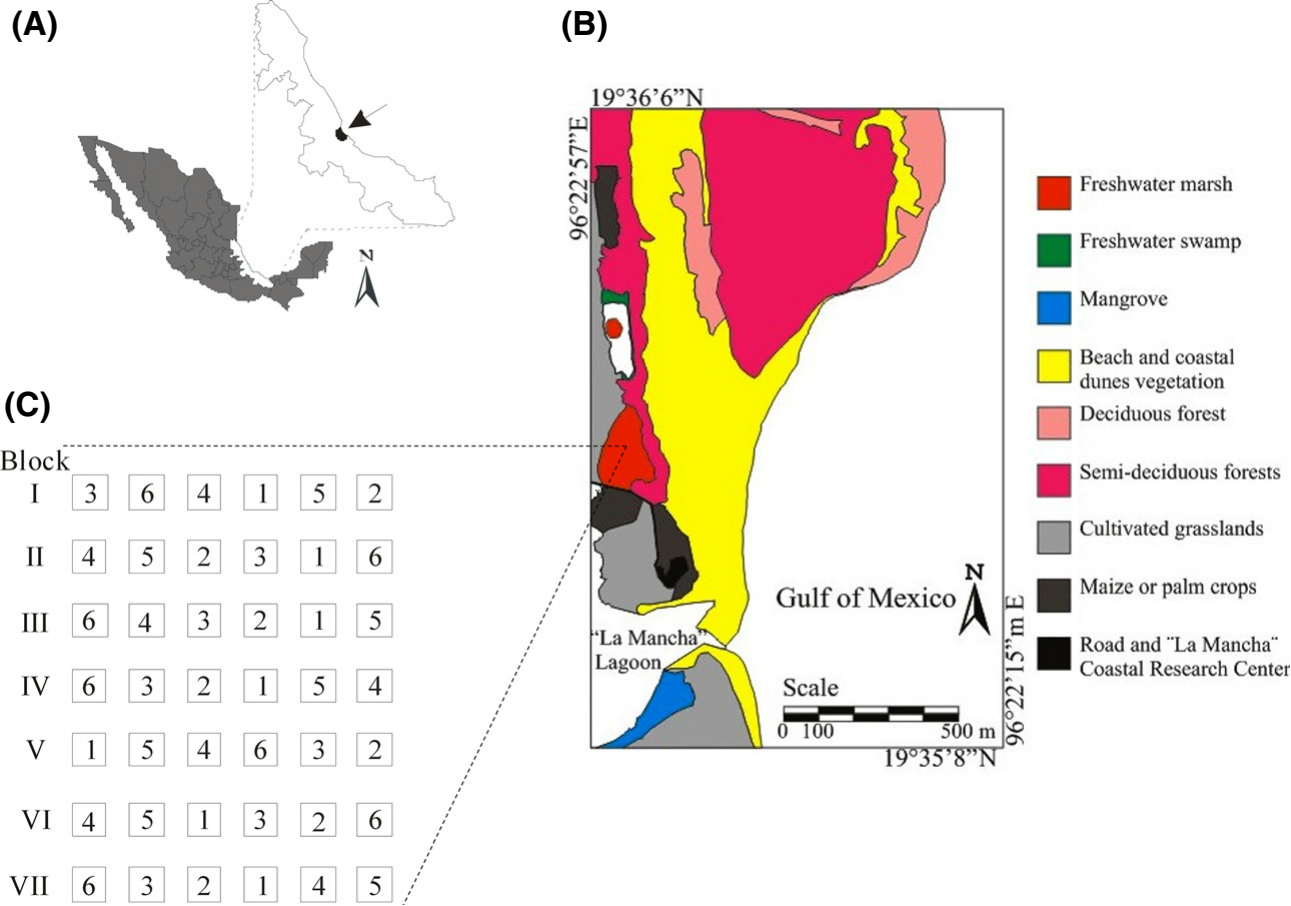
Location of the La Mancha study site in Veracruz, Mexico (A). Study site and surrounding vegetation types (B). Experimental design in the freshwater marsh (C). Roman numerals indicate blocks, and Arabic numbers indicate the treatment assigned to each plot.

Our field work was conducted in the La Mancha wetland (19°35′38″N, 96°22′53″W), a coastal freshwater marsh of ca. 3 ha, located in the central region of the state of Veracruz (Mexico), on the Gulf of Mexico (Fig.1A), close to the La Mancha lagoon (Fig.1B). The climate in this region is warm subhumid with a summer rainy season, and the mean annual precipitation is 1200–1500 mm. The mean annual temperature fluctuation is 22–26°C (Moreno-Casasola 2006). Novelo (1978) described the floristic composition of this wetland as an association of *Typha domingensis* Pers. mixed with other native species such as *Pontederia sagittata* C. Presl, *Sagittaria lancifolia* subsp. *media* (Micheli) Bogin, *Hydrocotyle umbellata* L., *Bacopa monnieri* (L.) Wettst., *Cyperus articulatus* L., *Crinum erubescens* Aiton, and *Limnocharis flava* (L.) Buchenau. Between 1978 and 1988, there was a project of integrated experimental farms in the wetland (Gómez-Pompa and Barrero Gámiz 1980). This project included nine chinampas (pre-Hispanic farming plots) over an area of 729 m^2^ with rice crops, fish-culture ponds, breeding fowl (ducks and hens), and anaerobic digesters for biogas production (López Martínez 1985; Travieso-Bello 2000). During this same period, a rancher introduced the African grass *E. pyramidalis* to an area contiguous with the experimental farm where cattle were grazed (A. Juárez pers. comm.). After 1988, these activities were abandoned, but cattle grazing continued on the adjacent land. Today, *E. pyramidalis* is found in the wetlands used for cattle grazing but not in upland pastures. After Novelo's work, in 1997 and 1999, two floristic studies (Travieso-Bello 2000; López Rosas et al. 2005) found that *E. pyramidalis* was the dominant species in the least flooded areas, while the most flooded areas were dominated by the native *S. lancifolia* or *T. domingensis*.

### Experimental design and data collection

We chose a 15 × 13 m rectangular area in the freshwater wetland, characterized by a homogeneous topography and the dominance of *E. pyramidalis*. There, we applied a randomized block experimental design with a random allocation of six treatment levels nested within each of seven replicate blocks. Each block measured 13 × 1 m and was separated from the others by a 1-m-wide buffer area. Each block was further divided into six 1 × 1 m permanent plots (experimental units) that were 1 m apart (Fig.1C). At the corners of each plot, we buried a 2.5-m aluminum rod to a depth of 0.8 m, leaving 1.7 m above the ground. In each plot, one of the following disturbance treatments was randomly applied. (1) *Control*: we left the plot in its natural state. (2) *Cut-transplant*: to give advantage over species with high ability of vegetative regeneration from roots, such as *E. pyramidalis*, we clipped all vegetation at soil level and planted 10 individuals of the native monocot *S. lancifolia* in the plot. We chose *S. lancifolia* (Alismataceae), an acaulescent C3 herb (max. height: 1.5 m) with wide leaves, and a thick, aerenchymatous petiole, because this is the dominant native species in the noninvaded marsh, and because this species can grow together with *E. pyramidalis* in invaded areas (López Rosas et al. 2005). For transplants, we selected individuals from the surrounding vegetation close to the experimental area. Before transplanting, each plant was trimmed to a standardized height of 50 cm. (3) *Shade*: to give advantage to native C3 species over the invader *E. pyramidalis*, with C4 photosynthesis, we kept a 1 × 1 × 1.7 m (side × side × height) shade cloth over the vegetation; we used shade mesh (no. 50) to reduce light intensity by 50%. We used shade mesh because it is a recurring practice in the control of invasive plants (Wheeler et al. 2011; Yang et al. 2014); there is experimental evidence that the exposure to the shade contributes to reduced photosynthetic capacity and light compensation in C4 African grasses (Dias-Filho 2002); and because the use of shade mesh has yielded positive results controlling other C4 species (Belsky 1994; Bunn et al. 1998; Cole and Weltzin 2005). (4) *Herbicide-shade*: to give advantage to native *S. lancifolia*, we placed all the individuals in a PVC tube covered with polythylene bags, and then we sprayed all of the unprotected vegetation of the plot with glyphosate (Round-Up™), a systemic herbicide that enters plants through the stomata and reaches the roots, competing with the essential aromatic amino acids and inhibiting protein formation (Franz et al. 1997). After spraying, we removed the PVC tube and plastic bags. We repeated this procedure 7 and 14 days after the first application to make sure we had removed all vegetation other than *S*. *lancifolia*. After the third application of the herbicide, we maintained all of the vegetation under shade cloth as described for treatment 3. (5) *Soil-transplant-shade*: we clipped all of the vegetation and then, to favor species that regenerate from the seed bank, we disked the soil with a shovel to a depth of 37 cm; we cut large rhizomes until we obtained a uniform, muddy consistency for the entire plot. Afterward, we transplanted 10 individuals of *S. lancifolia* as described for treatment 2, and then we maintained all of the vegetation under shade. (6) *Soil-transplant*: we proceeded as in treatment 5, except that the vegetation was not maintained under shade. Along the plot edges, including the *control*, we cut the roots and rhizomes with a shovel to a depth of 37 cm and sprayed herbicide in the buffer areas to prevent uncontrolled plant arrival from outside the plots. This procedure was repeated monthly until the end of the experiment.

We sampled all plots before beginning the treatments. Treatments were applied during the rainy season, and vegetation samples were collected 5, 7, 10, 15, and 19 months thereafter. From each sampling event, we recorded species composition and percentage cover per species each time (Kent 2011). Twenty months into the experiment, all plots were harvested to soil level; the vegetation on each was separated by species and oven-dried for 120 h at 65°C to obtain the dry aerial biomass of each species per plot.

When sampling the vegetation, we also measured water level, the electrical conductivity and pH of interstitial water, soil humidity and redox potential (Eh) of the soil. When the water table was below the soil surface, we obtained the water level using an *in situ* water sampler (McKee et al. 1988). The sampler was introduced slowly into the soil while aspirating with a plastic syringe until the water table had been reached. When the soil was flooded, we measured the water level directly with a ruler. Electrical conductivity and pH were measured from interstitial water samples from 15 cm below soil level, almost at the middle of the root zone. Eh was measured at 15 cm soil depth using three platinum electrodes, one calomel reference electrode (Corning 476340), and a digital pH/ORP Barnant meter. Platinum electrodes were calibrated in the laboratory with quinhydrone (Sigma Q-1001) in pH 4.0 buffer solutions (Bohn 1971). To calculate Eh, we added 244 mV to each mV reading (Patrick et al. 1996). We used the average of the three redox values for data analysis. Relative soil moisture was obtained by taking 15-cm-deep soil samples using aluminum boxes. For each sample, we recorded the wet weight and dry weight after oven-drying for 48 h at 80°C. Relative soil moisture results were obtained by subtracting dry weight from wet weight, dividing by wet weight and multiplying by 100.

### Data analysis

To get an overview of the growth of the invading species relative to the growth of native species, we calculated the “invader cover/native cover” ratio (cov*I*/cov*N*) for each sampling event by dividing the aboveground cover of the invasive grass by the aboveground cover of the native species (all species in the plot other than *E. pyramidalis*) in the same plot. At the end of the experiment, we calculated the “invader biomass/native biomass” ratio (biom*I*/biom*N*) for each plot by dividing the biomass of the invasive grass by the biomass of the native species. To avoid the error that would result from dividing by zero, we added a constant of 1 to all aboveground cover and biomass data.

We used the Shannon–Wiener function based on natural logarithm, *H*, as an index of species diversity (Peet 1974) to compare treatments at each sampling date. Shannon's equitability (*E*), that is, the evenness of allotment of individuals among species, was calculated as *H*/*H*max, where *H*max (maximum species diversity) = ln *S* (Peet 1974). For the five sampling dates mentioned above, we analyzed the cov*I*/cov*N* ratio, dominant species cover (i.e., those that had high cover and/or were relatively constant throughout the experiment), species richness (the number of species, *S*), *H* and *E* with a two-factor ANOVA with repeated measures (hereafter, RM-ANOVA) on both factors (von Ende 2001) to detect any differences (with *P*-value < 0.05), between treatments, sampling dates, and any interaction between the two. The biom*I*/biom*N* ratio and aboveground biomass of the dominant species in each treatment were also analyzed with a two-way ANOVA based on general linear model without interactions (Potvin 2001) to detect significant changes as a result of the treatment. Each of the physicochemical parameters recorded was compared with a two-factor RM-ANOVA to detect differences between treatments, sampling date, and the interaction between them.

Prior to the application of the analysis, we tested for assumptions of normality and homogeneity of variance in the response variables. To meet these assumptions, cov*I*/cov*N*, percentage cover, biom*I*/biom*N,* and relative soil moisture values were arcsine-square-root-transformed; richness was square root-transformed. When the statistical analysis showed a significant effect due to the explanatory variables, we compared the means of the response variables with a *post hoc* SNK multiple comparison test. All statistical analyses were run in Statistica 8.0 software (StatSoft, Inc., Tulsa, OK).

In order to detect main variation trends in the effect of treatments on the plots before the experiment started and at the end of it, we applied an ordination using nonmetric multidimensional scaling (NMDS; Faith et al. 1987; Minchin 1987). This was done with a biotic matrix (84 plots −42 from rainy season before the experiment was started and 42 from rainy season 15 months after treatments were applied × 20 species) that included percent cover data transformed by square root to reduce the influence of dominant species; an abiotic matrix, including standardized environmental characteristics (84 plots × 5 environmental characteristics) that included values of the physico-chemical parameters before the experiment was started and before harvest (water level, interstitial pH and conductivity, and soil Eh and moisture). NMDS was done using the Sørensen index as a distance measure between plots, a stability criterion of 1 × 10^−5^ and a maximum of 200 iterations. The program used was PC-ORD, version 4.25 (McCune and Grace 2002).

To analyze the relationships between plots, we used the two matrices (biotic and abiotic) listed above and subjected them to hierarchical cluster, which helped us to group the plots, based on the Sørensen similarity index. In order to detect differences between groups of plots, we applied an analysis of similarities (ANOSIM, Clarke and Green 1988) based on permutation/randomization methods on the resemblance matrix. To determine the prevalent plant species in each group, we described the significant groups derived from ANOSIM by applying a similarity percentage analysis—species contributions (SIMPER, Clarke 1993). To determine the environmental variables that best explain the observed plot groups based on the plant composition, we applied the BEST procedure (Clarke et al. 2008) as an indirect gradient analysis. This last analysis used the Spearman rank correlation method and a resemblance matrix based on the Euclidian distance between pairs of plots based on the normalized (standardized) environmental characteristics.

## Results

The dominant species throughout the experiment were the invasive grass *E. pyramidalis* (Poaceae) and the native hydrophytes *S. lancifolia* (Alismataceae), *Pontederia sagittata* (Pontederiaceae), *Fuirena simplex* Vahl (Cyperaceae), and *Hydrocotyle umbellata* (Apiaceae).

### Invader: natives ratios, species cover, and biomass

**Figure 2:**
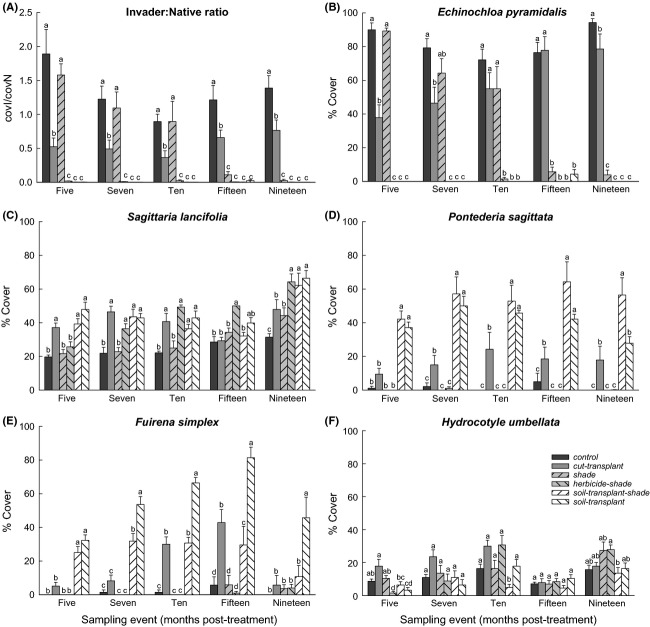
Ratio response for cov*I*/cov*N* and the percent cover response (mean + 1SE; *n *=* *7) of five dominant hydrophytes over time in six treatments. Different letters indicate significant differences among means for each sampling date (two-way RM-ANOVA; *P *<* *0.05). For the analysis, all data were arcsine-square-root-transformed.

**Figure 3:**
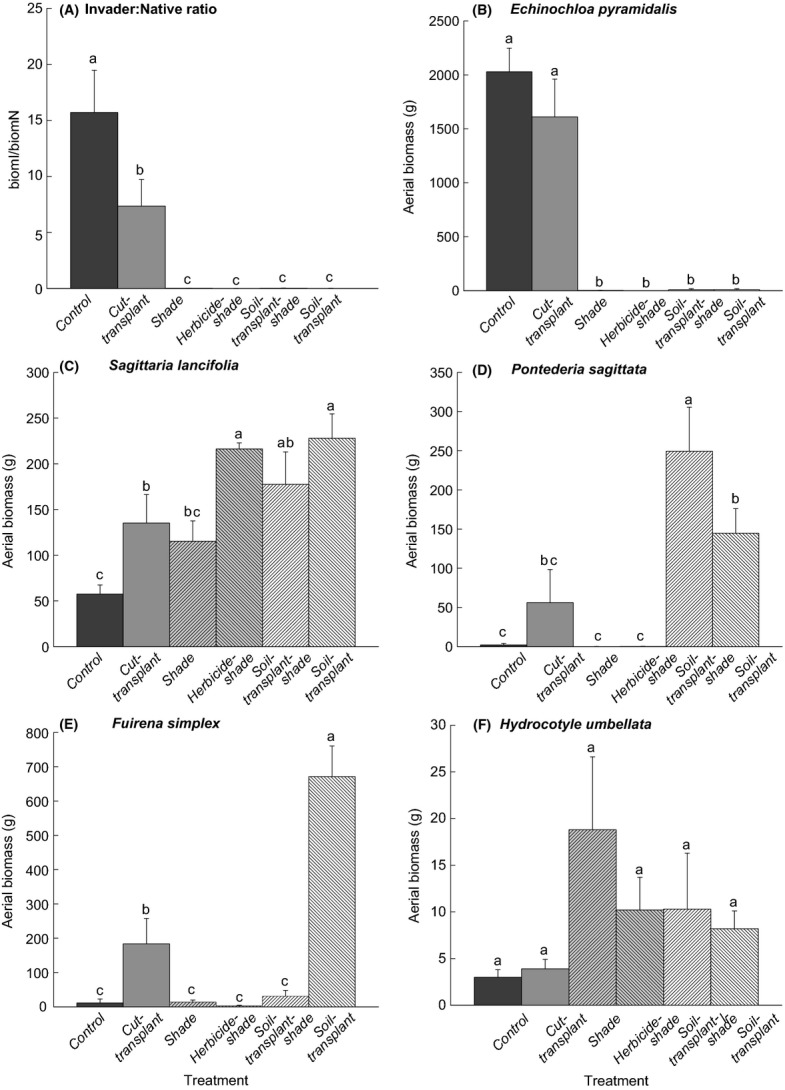
Ratio response for biom*I*/biom*N* and aerial biomass (mean + 1SE; *n *=* *7) for five dominant hydrophytes under six different treatments in the La Mancha freshwater wetland. Harvesting took place in dry season, 19 months after the treatments were applied. Different letters indicate significant differences among means (two-way ANOVA; *P *<* *0.05).

The RM-ANOVA detected significant sampling date × treatment interactions for the cov*I*/cov*N* ratio (*F*_20,120_ = 16.1; *P *<* *0.001) and the aerial cover of the five dominant species (*F*_20,120_ = 27.8, 4.9, 2.2, 5.4, and 4.2; *P *<* *0.001, 0.001, 0.01, 0.001, and 0.001, respectively for *E. pyramidalis*, *S. lancifolia*, *P. sagittata*, *F. simplex*, and *H. umbellata*). The ANOVA also detected significant differences in the effect of the treatment on biom*I*/biom*N* (*F*_5,30_ = 27.9; *P *<* *0.001) and the aerial biomass of *E. pyramidalis*, *S. lancifolia*, *P. sagittata*, and *F. simplex* (*F*_5,30_ = 27.8, 7.4, 10.7, and 33.6, respectively; all with *P *<* *0.001), but not on the aerial biomass of *H. umbellata* (*F*_5,30_ = 1.7; *P > *0.05). ANOVA tables are presented in the Appendix S1. In the *herbicide-shade*, *soil-transplant-shade*, and *soil-transplant* treatments, the cov*I*/cov*N* ratios were always the lowest, and significantly so, during the experiment (Fig.2A). In the *shade* treatment, this ratio was significantly higher from the fifth to the tenth month post-treatment, but not different with respect to the *control*, and decreased to the lowest values in the fifteenth month post-treatment, without recovering by the end of the experiment. The *control* had the highest ratios throughout the experiment, followed by those of the *cut-transplant* treatment (Fig.2A). The highest biom*I*/biom*N* ratio was obtained for the *control*, followed by intermediate values in the *cut-transplant* treatment, and the lowest ratios were obtained for the *shade*, *herbicide-shade*, *soil-transplant-shade*, and *soil-transplant* treatments (Fig.3A).

A similar pattern was observed for the aerial cover and aerial biomass of *E. pyramidalis* (Figs2B and 3B). *Sagittaria lancifolia* was present on all sampling dates with relatively high cover (19–66%) and tended to increase toward the end of the experiment in all treatments (Fig.2C). Throughout the experiment, the cover of this species was closely related to the behavior of *E. pyramidalis*. *Sagittaria lancifolia* had the lowest cover values in the *control*, where *E. pyramidalis* dominated, whereas in the *herbicide-shade*, *soil-transplant-shade*, and *soil-transplant* treatments, which succeeded in eliminating the invasive species, *S. lancifolia* had significantly greater cover. At the end of the experiment, the aerial biomass of *S. lancifolia* was significantly higher in the *soil-transplant*, *herbicide-shade*, and *soil-transplant-shade* treatments; values were intermediate in the *cut-transplant* and *shade* treatments, and the lowest value was obtained for the *control* (Fig.3C). *Pontederia sagittata* had the highest cover values in the *soil-transplant-shade* and *soil-transplant* treatments, intermediate values in the *cut-transplant* treatment, and the lowest values in the *control* and in the *shade* and *herbicide-shade* treatments (Fig.2D). At the end of the experiment, there were no significant differences between the cover in the *soil-transplant* treatment and that of the *cut-transplant* treatment. The highest biomass for this species was recorded for the *soil-transplant-shade* treatment, intermediate values for the *soil-transplant* and *cut-transplant* treatments, and the lowest values for the *control* and *shade* and *herbicide-shade* treatments (Fig.3D). *Fuirena simplex* had the significantly highest values in the *soil-transplant* treatment in both cover values throughout the experiment (Fig.2E) and the greatest biomass at the end of experiment (Fig.3E). The cover value for this species was significantly higher in the *soil-transplant-shade* treatment, but gradually decreased to lower values at the end of the experiment. In the *shade* treatment, cover for *F. simplex* was low in the fifth month post-treatment, but gradually increased to intermediate values in the tenth and fifteenth months post-treatment and then decreased again to low values in the nineteenth month post-treatment. The lowest cover and biomass values for this species were recorded for the *control*, in *herbicide-shade* and *soil-transplant-shade* treatments (Figs2E and 3E). *Hydrocotyle umbellata* was present on all sampling dates in all treatments but with relatively low cover (<30%) that differed slightly among treatments in the fifth, tenth, and nineteenth months post-treatment (Fig.2F). At the end of the experiment, its aerial biomass did not differ between treatments (Fig.3F).

### Species richness and diversity

The RM-ANOVA detected significant sampling date × treatment interactions for all diversity indicators (*F*_20, 120_ = 5.2, 5.6, and 4.6 for *S*, *H* and *E,* respectively; all with *P *<* *0.001; Table1). ANOVA tables are presented in the Appendix S1. From the fifth to the seventh month post-treatment, species richness (*S*) and *H* values were higher in the *cut-transplant* and *soil-transplant* treatments; values were intermediate or low in the *control*, *shade,* and *soil-transplant-shade* treatments, and the values were lowest in the *herbicide-shade* treatment. At the end of the experiment, in the nineteenth month post-treatment, *S* and *H* values did not differ among treatments. In the fifth and the seventh month post-treatment, the equitability values (*E*) were low in the *herbicide-shade* treatment, but treatment had no significant effect on the last three sampling dates (from the tenth to the nineteenth months post-treatment; Table1).

**Table 1:** Species richness (*S*), diversity (*H*), and equitability (*E*) under different disturbance treatments on five sampling events. Means ± 1 standard error are shown for the seven replicate plots

	Treatment					
	Control	Cut-transplant	Shade	Herbicide-shade	Soil-transplant-shade	Soil-transplant
5 Months post-treatment (*nortes* season)						
Richness (*S*)	5.0 ± 0.3 a	5.9 ± 0.6 a	4.7 ± 0.4 a	2.1 ± 0.5 b	4.9 ± 0.1 a	4.4 ± 0.4 a
Diversity (*H*)	1.13 ± 0.10 a	1.42 ± 0.09 a	1.14 ± 0.07 a	0.38 ± 0.16 b	1.34 ± 0.04 a	1.28 ± 0.06 a
Equitability (*E*)	0.70 ± 0.04 b	0.82 ± 0.02 ab	0.74 ± 0.02 ab	0.34 ± 0.13 c	0.85 ± 0.02 ab	0.88 ± 0.02 a
7 Months post-treatment (dry season)						
Richness (*S*)	5.3 ± 0.3 ab	6.4 ± 0.9 a	4.1 ± 0.4 b	2.9 ± 0.6 c	6.4 ± 0.6 a	6.6 ± 0.5 a
Diversity (*H*)	1.30 ± 0.07 ab	1.54 ± 0.13 ab	1.22 ± 0.12 b	0.68 ± 0.18 c	1.55 ± 0.09 ab	1.66 ± 0.03 a
Equitability (*E*)	0.79 ± 0.03 a	0.87 ± 0.04 a	0.87 ± 0.03 a	0.64 ± 0.12 b	0.85 ± 0.01 a	0.89 ± 0.02 a
10 Months post-treatment (rainy season)						
Richness (*S*)	5.4 ± 0.3 b	7.9 ± 0.9 a	4.9 ± 0.3 b	3.1 ± 0.4 c	5.4 ± 0.4 b	8.0 ± 0.3 a
Diversity (*H*)	1.45 ± 0.06 b	1.89 ± 0.11 a	1.35 ± 0.10 b	0.96 ± 0.10 b	1.48 ± 0.09 b	1.91 ± 0.03 a
Equitability (*E*)	0.86 ± 0.02 a	0.93 ± 0.01 a	0.85 ± 0.04 a	0.89 ± 0.04 a	0.88 ± 0.02 a	0.92 ± 0.003 a
15 Months post-treatment (rainy season)						
Richness (*S*)	5.3 ± 0.4 b	8.0 ± 0.4 a	3.6 ± 0.3 c	3.7 ± 0.3 c	4.9 ± 0.4 bc	6.4 ± 0.6 b
Diversity (*H*)	1.27 ± 0.09 bc	1.66 ± 0.04 a	1.03 ± 0.06 c	0.97 ± 0.08 c	1.16 ± 0.12 c	1.50 ± 0.09 ab
Equitability (*E*)	0.77 ± 0.04 a	0.80 ± 0.01 a	0.83 ± 0.04 a	0.75 ± 0.04 a	0.73 ± 0.05 a	0.82 ± 0.01 a
19 Months post-treatment (dry season)						
Richness (*S*)	5.0 ± 0.4 a	5.9 ± 0.5 a	5.0 ± 0.7 a	5.0 ± 0.5 a	5.0 ± 0.4 a	6.3 ± 0.4 a
Diversity (*H*)	1.22 ± 0.08 a	1.47 ± 0.08 a	1.41 ± 0.13 a	1.33 ± 0.09 a	1.29 ± 0.09 a	1.61 ± 0.05 a
Equitability (*E*)	0.76 ± 0.02 a	0.84 ± 0.02 a	0.91 ± 0.03 a	0.85 ± 0.03 a	0.81 ± 0.02 a	0.88 ± 0.01 a

Different letters between columns indicate significant differences (two-way RM-ANOVA, *P *<* *0.05). For the purposes of analysis, species richness data were square root-transformed.

### Physicochemical parameters

The pH and the conductivity of the interstitial water and soil moisture were not significantly affected by any of the disturbance treatments (*F*_5,30_ = 0.69, 1.66, and 0.91, respectively; all with *P *>* *0.05), and there were no treatment × sampling date interactions (*F*_20,120_ = 1.26, 1.45, and 0.96, respectively; all with *P *>* *0.05). ANOVA tables are presented in the Appendix S1. These three parameters changed significantly over time: pH values increased from the fifth to seventh and tenth months post-treatment, and then decreased in the fifth and nineteenth months post-treatment (Table2). Electrical conductivity was relatively stable over time, decreasing only slightly in tenth and nineteenth months post-treatment. Soil moisture increased from the fifth to seventh and tenth months post-treatment, and then increased in the fifteenth month post-treatment, but decreased to a level similar to that of the fifth month in the nineteenth month post-treatment (Table2). For water level and soil Eh values, we detected significant sampling date × treatment interactions (*F*_20,120_ = 2.51 and 1.80; *P *<* *0.01 and 0.05, respectively). ANOVA tables are presented in the Appendix S1. In fifth, seventh, and nineteenth months post-treatment, the water level was below ground level (negative values) and was not different among treatments (Table3). In the tenth month post-treatment, there were the driest environmental conditions, and the significantly drier treatments were the *shade*, *herbicide-shade,* and the *control*. In the fifth month post-treatment, the water level rose and the levels were significantly lower in the *control* and *shade* treatments. In the nineteenth month post-treatment, the water level decreased again but was not different among treatments (Table3). Soil Eh was closely related to water level. When the water level dropped, Eh increased and vice versa. In the fifth month post-treatment, the highest Eh value was recorded for the *soil-transplant* treatment, intermediate values for the *control,* the *cut-transplant*, *shade,* and *soil-transplant-shade* treatments, with a significantly lower value for the *herbicide-shade* treatment (Table3). In the fifth and seventh months post-treatment, Eh was not affected by the treatments. In the fifth month post-treatment, Eh was highest in the *control*, values were intermediate in the *shade*, *herbicide-shade* and *soil-transplant* treatments, and significantly lower values were recorded for the *cut-transplant* and *soil-transplant-shade* treatments. In the nineteenth month post-treatment, Eh increased with respect to the fifteenth month, but was not affected by the treatments (Table3).

**Table 2:** Interstitial pH, electric conductivity and soil moisture on five sampling events in the wetland of La Mancha, Veracruz. Means ± 1 SE are shown for 42 experimental plots (6 treatments × 7 blocks)

	Sampling event (months post-treatment)/climatic season					*F*_4,24_
	Five/*nortes*	Seven/dry	Ten/rainy	Fifteen/rainy	Nineteen/dry	
pH	6.4 ± 0.02 c	7.5 ± 0.05 a	7.7 ± 0.03 a	7.0 ± 0.02 b	7.1 ± 0.02 b	76.6***
Conductivity (mS/cm)	0.93 ± 0.01 a	0.92 ± 0.01 a	0.87 ± 0.01 b	0.91 ± 0.01 a	0.89 ± 0.01 ab	5.4**
Soil moisture (%)	63.2 ± 0.56 b	58.9 ± 0.68 c	59.1 ± 0.67 c	65.8 ± 0.56 a	61.9 ± 0.78 b	17.4***

Different letters between columns indicate significant differences (two-way RM-ANOVA

** *P *<* *0.01

*** *P *<* *0.001). For the purposes of analysis, soil moisture data were arcsine square root-transformed.

**Table 3:** Water level and soil Eh under different disturbance treatments on five sampling events in the wetland of La Mancha, Veracruz. Means ± 1 SE are shown for seven replicate plots

	Treatment					
	Control	Cut-transplant	Shade	Herbicide-shade	Soil-transplant-shade	Soil-transplant
5 Months post-treatment (*nortes* season)						
Water level (cm)	−5.4 ± 0.4 a	−4.9 ± 0.6 a	−5.33 ± 0.6 a	−5.1 ± 0.6 a	−5.4 ± 0.6 a	−4.3 ± 0.3 a
Eh (mV)	109.5 ± 7.6 ab	116.3 ± 4.8 ab	106.8 ± 5.4 ab	94.2 ± 4.5 b	113.3 ± 5.8 ab	126.3 ± 6.9 a
7 Months post-treatment (dry season)						
Water level (cm)	−6.4 ± 0.6 a	−6.3 ± 0.5 a	−6.7 ± 0.5 a	−6.4 ± 0.4 a	−6.4 ± 0.4 a	−6.1 ± 0.5 a
Eh (mV)	143.1 ± 7.8 a	153.5 ± 7.6 a	143.0 ± 6.8 a	143.9 ± 12.9 a	152.3 ± 9.1 a	154.1 ± 6.5 a
10 Months post-treatment (rainy season)						
Water level (cm)	−13.7 ± 0.7 b	−11.3 ± 1.0 a	−14.7 ± 0.6 b	−13.4 ± 0.9 b	−10.4 ± 1.1 a	−11.1 ± 0.9 a
Eh (mV)	378.3 ± 4.0 a	375.2 ± 2.0 a	381.3 ± 3.7 a	376.7 ± 3.6 a	376.3 ± 3.8 a	372.5 ± 5.3 a
15 Months post-treatment (rainy season)						
Water level (cm)	−2.7 ± 0.6 b	−1.0 ± 0.6 ab	−1.0 ± 0.8 b	0.4 ± 0.5 a	1.1 ± 0.5 a	−0.7 ± 0.6 ab
Eh (mV)	136.7 ± 13.5 a	113.3 ± 10.5 b	115.7 ± 9.4 ab	131.3 ± 10.3 ab	111.4 ± 8.8 b	127.4 ± 14.8 ab
19 Months post-treatment (dry season)						
Water level (cm)	−7.3 ± 0.6 a	−6.6 ± 0.4 a	−6.4 ± 0.3 a	−6.4 ± 0.6 a	−6.2 ± 0.4 a	−6.3 ± 0.7 a
Eh (mV)	141.3 ± 4.8 a	136.3 ± 5.5 a	127.5 ± 3.1 a	135.3 ± 9.8 a	133.0 ± 6.0 a	149.7 ± 2.8 a

Different letters between columns indicate significant differences. There was a significant sampling event × treatment interaction in water level (*F*_20,120_ = 2.5, *P *=* *0.001) and Eh (*F*_20,120_ = 1.8, *P *<* *0.05).

### Multivariate analysis

**Figure 4:**
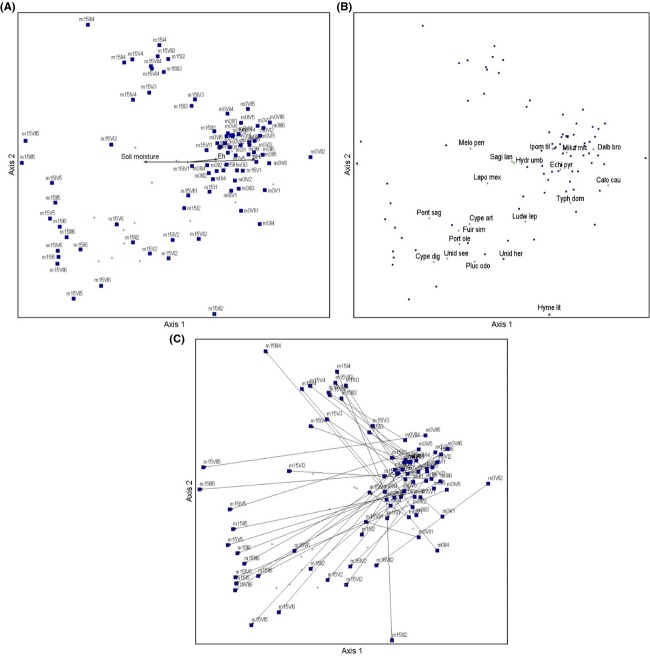
NMDS ordination of 84 plots (42 from rainy season before the experiment was started –m0–, and 42 from rainy season 15 months after treatments were applied –m15–) and plant species (stress = 13.27 for two dimensions; final instability = 0 with 61 iterations). (A) The ordination shows a gradient from the plots with elevate values of pH and low values of soil moisture on the center-right side of the ordination space to low values of pH and high values of soil moisture in left side. (B) Species with the highest negative correlations along Axis 1 are *Pontederia sagittata* and the sedges *Fuirena simplex*, *Cyperus digitatus* and *C. articulatus*; the highest positive values along Axis 1 were found for *Dalbergia brownei*, *Echinochloa pyramidalis* and *Typha domingensis*. On Axis 2, the highest positive correlations were found for *Dalbergia brownei*, *Echinochloa pyramidalis* and the vines *Ipomoea tiliacea* and *Mikania micrantha*. At the other extreme are *Hymenocallis littoralis* and *Pluchea odorata*. (C) Arrows join sampling during m0 and m15 for each plot; in the center-right space of ordination are plots with high cover of *Echinochloa pyramidalis* (all m0 plots, the seven control plots from m15 and the seven plots from m15 subjected to cut-transplanted treatments).

The two-dimensional NMDS ordination of 84 plots and plant species (stress = 13.27 for two dimensions; final instability = 0 with 61 iterations) shows a gradient from the plots with elevate values of pH and low values of soil moisture on the center-right side of the ordination space to low values of pH and high values of soil moisture in left side (Fig.4A). In this gradient, *E. pyramidalis* is more abundant on the center-right side of the ordination space that includes plots with vegetation sampled in pretreatments conditions (Month 0 –m0), and plots from preharvest conditions (Month 15 –m15) corresponding to the control and cut-transplant treatment (Fig.4B). On the left side of the ordinations space, there are plots from m15, characterized by scarce or null presence of *E. pyramidalis* and a greater hydrophytes richness (Fig.4B). Figure4C presents the same ordinations where the plots in m0 and m15 are joined by successional vectors. Most of the plots migrate to the left side. Plots with high cover of *Pontederia sagittata* and the sedges *Fuirena simplex* and *Cyperus digitatus* are at the bottom-left side of the graph. The Pearson correlations (*r*) between each environmental characteristic or each species and both NMDS axes are presented in Tables4 and 5, respectively.

**Table 4:** Pearson correlations (*r*) of environmental characteristics with the two NMDS axes of the ordination in Figure4

Physicochemical characteristics	NMDS axis	
	1	2
pH	0.684	0.128
Conductivity	0.051	−0.005
Eh	0.458	0.145
Water level	−0.415	0.030
Soil moisture	−0.551	0.053

**Table 5:** Pearson correlations (*r*) of species with the two NMDS axes of ordination in Figure4

Species	NMDS axes	
	1	2
*Calopogonium caeruleum* (Benth.) Hemsley	0.182	−0.055
*Cyperus articulatus* L.	−0.113	−0.152
*Cyperus digitatus* Roxb.	−0.283	−0.369
*Dalbergia brownei* (Jacq.) Urban	0.458	0.103
*Echinochloa pyramidalis* (Lam.) Hitchc. & A. Chase	0.830	−0.166
*Fuirena simplex* Vahl	−0.619	−0.766
*Hydrocotyle umbellata* L.	−0.229	0.006
*Hymenocallis litoralis* (Jacq.) Salisb.	0.042	−0.280
*Ipomoea tiliacea* (Willd.) Choisy	0.800	0.441
*Laportea mexicana* (Liebm.) Wedd.	−0.218	−0.146
*Ludwigia octovalvis* (Jacq.) Raven	0.006	−0.152
*Melothria pendula* L.	−0.175	0.057
*Mikania micrantha* Kunth	0.346	−0.098
*Pluchea odorata* (L.) Cass.	−0.128	−0.307
*Pontederia sagittata* C. Presl	−0.750	−0.519
*Portulaca oleracea* L.	−0.358	−0.537
*Sagittaria lancifolia* L. subsp. *media* (Michelin) Bogin	−0.658	0.099
*Typha domingensis* Pers.	0.153	−0.150
Unidentified herb (Umbelliferae)	−0.030	−0.177
Unidentified seedling	−0.311	−0.460

**Figure 5:**
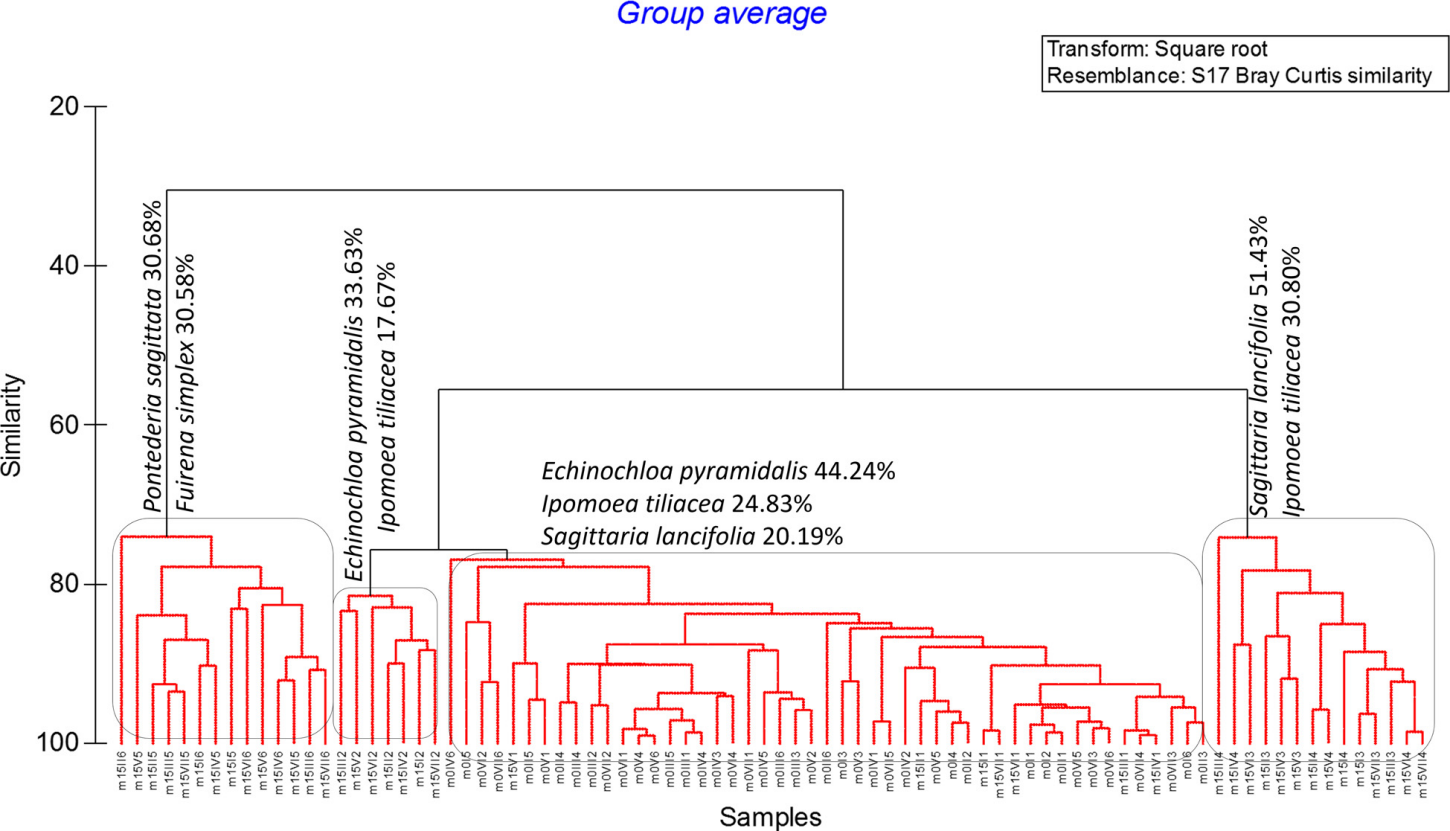
Numerical cluster of 84 plots (42 from rainy season before the experiment was started –m0–, and 42 from rainy season 15 months after treatments were applied –m15–) based on the percent cover of plant species. Rounded rectangles indicate significant differences between groups (SIMPROF analysis, *P *<* *0.05). From left to right, the first group includes plots from m15 subjected to soil-transplant-shade and soil-transplant treatments. The second group includes the seven plots from m15 subjected to cut-transplanted treatments. The third group contains all the plots from m0 and the seven control plots from m15. The fourth group is composed by all the plots from m15 subjected to shade and herbicide-shade treatments.

According to the plant species composition, most plots cluster into four significant groups (rounded rectangles in Fig.5; SIMPROF analysis; *π *= 7.959, *P *=* *0.001). Scanning from left to right groups in Figure5, the first group had an average similarity 80.14% and includes plots from m15 subjected to soil-transplant-shade and soil-transplant treatments. Those plots are characterized by a dominance of *Pontederia sagittata* (that contributed with 30.68%) and the sedge *Fuirena simplex* (with 30.58% contribution). The second group had an average similarity 83.54% and includes the seven plots from m15 subjected to cut-transplanted treatments. Those plots are characterized by a dominance of *Echinochloa pyramidalis* (that contributed with 33.63%) and the presence of the vine *Ipomoea tiliacea* (with 17.67% contribution). The third group is the biggest, and it contains all the plots from m0 and the seven control plots from m15. This group is characterized by a high dominance of *E. pyramidalis* (that contributed with 44.24%), which is accompanied by *I. tiliacea* (contribution of 24.83%) and *S. lancifolia* (contribution of 20.19%). The fourth group is composed by all the plots from m15 subjected to shade and herbicide-shade treatments. Those plots are characterized by a dominance of *Sagittaria lancifolia* (that contributed with 51.43%), accompanied by the vine *I. tiliacea* (contribution of 30.80%). The BEST analysis indicates that overall the environmental parameters that best explained the assemblage of species in plots was a combination of interstitial pH, water level, and soil moisture (BEST/BIOENV; ρ = 0.303, *P *=* *0.01).

## Discussion

The results of this study and previous research in this ecosystem (López Rosas et al. 2006, 2010) suggest that it is possible to restore freshwater wetlands that are heavily invaded by grasses. In the control quadrats, *E. pyramidalis* was dominant over all the other species, as reflected by the low values of diversity, equitability and the invader:natives ratios. This is consistent with the findings of previous research (López Rosas et al. 2006; López Rosas and Moreno-Casasola 2012) and indicates that neither the recovery of native vegetation nor the reduction of the invasion would be expected as natural outcomes in the short term.

Low-intensity disturbances, such as cutting, are not efficient at eliminating *E. pyramidalis*, but do positively affect the germination of some native species. The *cut-transplant* treatment caused an initial decline in *E. pyramidalis* cover and an increase in the abundance of the seedlings of native species such as *P. sagittata* and the sedges *F. simplex*, *Cyperus digitatus*, and *Eleocharis geniculata*. This, together with the re-introduction of *S. lancifolia* via transplant, contributed to the increase in the diversity indicators and the decrease of the cov*I*/cov*N* ratio for the first sampling dates relative to the *control*. These changes peaked in the tenth month post-treatment, after which they decreased to levels very similar to those of the *control*. This was due to the gradual recovery of *E. pyramidalis*, for which high cover values were recorded at the end of the experiment; recovery that was successful to the extent that its biomass for this treatment was not different from the *control* and was significantly higher than in the other treatments. The presence of seedlings of various species, among them several Cyperaceae, may have resulted from the initial cutting of vegetation allowing light to reach ground level, which stimulated the germination from the seed bank. Removing vegetation to leave the soil bare invariably results in the germination of these species (López Rosas et al. 2006, 2010), indicating their role as pioneers in the process of ecological succession. The absence of these species before cutting the vegetation indicates that their occurrence is through germination of the seed bank and not by regeneration from roots or rhizomes. On the other hand, the recovery of *E. pyramidalis* after cutting is not unexpected because it is adapted to foraging. The grasses that are adapted to foraging are not killed by defoliation; they are able to resprout from rhizomes or stem fragments, and in some cases, growth is actually stimulated by defoliation (D'Antonio and Vitousek 1992; Baruch and Jackson 2005; Wang et al. 2009; Sasaki et al. 2011).

With the *shade* treatment, we were able to eliminate the invader after 19 months. The process was gradual and hardly perceptible in the first sampling, but by the fifteenth month post-treatment, the invader:natives ratio had decreased significantly due to the death of *E. pyramidalis* and the survival of native species, and stayed low until the end of the experiment. The diversity indicators in this treatment did not increase with the removal of the invader; however, they came to have very low values, similar to those of the *control*. In this treatment, *S. lancifolia* maintained its cover and biomass levels, also similar to those of the *control*. The response of these two species to this treatment reinforces the hypothesis proposed by López Rosas et al. (2005) that their co-existence in the wetland is the result of their different photosynthetic pathways. *Sagittaria lancifolia*, a species with C3 photosynthesis, has advantages over *E. pyramidalis*, a species with C4 photosynthesis, during the winter when there is less light and temperatures are lower, while the opposite occurs in the summer, demonstrating a complementarity of resource use (Díaz and Cabido 2001). These temporal changes are reflected in differences in biomass production of both species. *Sagittaria lancifolia* is more productive in winter, and *E. pyramidalis* in the summer (López Rosas et al. 2005). On the other hand, the fact that we succeeded in eliminating the invader by the end of the experiment, but not in increasing the richness or diversity of native species, suggests that shade is a strong inhibitor of seed bank germination. This result indicates that the extensive cover of *E. pyramidalis* in the wetland decreased light penetration to ground level, preventing the germination of native species. This is reinforced by the results for the last three treatments. These findings are consistent with the statement of D'Antonio et al. (2011) indicating that “where invaders have become dominant after a disturbance, they are more likely to remain dominant if they establish conditions that interfere with recruitment of native species even if seed sources of the latter are readily available.” To determine whether *E. pyramidalis* is acting as a “driver” or “passenger” (MacDougall and Turkington 2005; HilleRisLambers et al. 2010), will be necessary to do new experimental tests with plots planted with the invasive species together with control plots with both undisturbed and disturbed native vegetation, and assess whether the invasive grows toward both types of plots (driver) or only to the disturbed plots (passenger). By the way we design our experiment, we cannot confirm whether *E. pyramidalis* is acting as a passenger or driver because we made maintenance of experimental plots so that we do not allow the entry of roots nor rhizomes of the invading in the first 37 cm depth of soil.

The *herbicide-shade* treatment killed all of the species except *S. lancifolia,* which we had protected from the herbicide. The invader failed to recover during the 19 months of the experiment. Unlike the *shade* treatment, the *herbicide-shade* treatment produced a significant increase in the cover and biomass of *S. lancifolia*. This was due to this species' tolerance to shade and the increased availability of space resulting from the death of the dominant species at the beginning of the treatment. This suggests that, had the *shade* treatment been continued over a longer period of time, the cover of *S. lancifolia* would have increased. On the other hand, the low cover of other native species in the *herbicide-shade* treatment confirms that shade inhibits the germination of many wetland species.

The most successful treatments in the elimination of the invader and the recovery of native species in space and time were those in which the soil was disked at the beginning of the experiment: the *soil-transplant-shade* and s*oil-transplant* treatments. In both of these, the invader:natives ratios were lowest, and they had the highest values for the diversity indicators; these values were constant throughout the experiment. In both treatments, germination from the seed bank was high at the start of the experiment as a result of increased light reaching the soil when the vegetation was cut to ground level, but this only occurred for about a week before the shade mesh was placed over the *soil-transplant-shade* treatment. This prevented the other seeds from germinating, and inhibited the growth of light-demanding species. In these treatments, as in the *cut-transplant* treatment, the species that germinated at the beginning of the experiment were mainly *P. sagittata* and the sedges *F. simplex*, *C. digitatus,* and *E. geniculata*. However, in the *soil-transplant-shade* treatment, the species with the greatest cover and biomass at the end of the experiment were *S. lancifolia* and *P. sagittata*, while for the *soil-transplant* treatment values were highest for *S. lancifolia* and *F. simplex*. Based on this, it is difficult to say whether the differences in vegetation response between treatments were due solely to differences in light tolerance between the two groups of species or whether competition also played a role in reducing *P. sagittata* in the *soil-transplant-shade* treatment and *F. simplex* in the *soil-transplant* treatment. To address this, we would need to know more about the types of photosynthesis of *P. sagittata* and the sedges present in this study, or alternatively, conduct an experiment with exclusion treatments, exposing sedges to bright environments, excluding *P. sagittata* from shaded environments and excluding *S. lancifolia* from both light conditions. Soil disking, without transplanting *S. lancifolia*, led to the germination of the seed bank of many native species (López Rosas et al. 2006), but these species were not competitive enough to prevent the reinvasion by *E. pyramidalis*. Similarly, Lindig-Cisneros and Zedler (2002) experimentally increased the density of key native species in areas where the invasive grass *Phalaris arundinacea* had previously been removed, thus generating an increase in cover that inhibited the germination of the invader.

After an overall analysis of the effects of the different treatments on vegetation, we can conclude that the high cover and biomass production of *E. pyramidalis* in the wetlands are favored by its C4 photosynthesis and its high capacity for vegetative propagation by rhizomes and stems in a warm climate with an unlimited water supply. Once established, this grass can survive and reproduce under drier conditions. The high cover of this invader inhibits the germination of most native wetland species. The exclusion of native vegetation coupled with the thick growth of the grass contributes to its success as an invader. As suggested by López Rosas et al. (2006), to eliminate the invader and restore the native vegetation of the wetland, it is necessary to destroy underground structures of *E. pyramidalis,* to both prevent vegetative growth and also create conditions for the germination of the seed bank. A useful contribution of this study to the previous proposal is the use of shade mesh, with the advantage that it can be installed on a large scale at a relatively low cost, compared to the economic and ecological impact involved in disking large areas of soil. In controlled treatments (López Rosas et al. 2010), there is no evidence of *E. pyramidalis* regrowth after killed by prolonged exposure to the shade. Shading would contribute to the elimination of the invader in the first stage of restoration, and with monitoring it would be possible to assess whether the system will shift into a passive restoration process or whether, as suggested by Osland (2009) and Middleton et al. (2010), it is necessary to select and plant native species that are able to compete with invading species and resist invasion.

## Supporting Information

**Appendix S1.** ANOVA tables for cov*I*/cov*N,* percent cover of five dominant hydrophytes; biom*I*/biom*N*, aerial biomass of five dominant hydrophytes; diversity indicators and physicochemical characteristics mentioned in the Results section. **Table S1.** Repeated measures ANOVA table for the ratio response for cov*I*/cov*N* and the percent cover response of five dominant hydrophytes over time in six treatments. **Table S2.** Two-way ANOVA table for the ratio response for biom*I*/biom*N* and aerial biomass for five dominant hydrophytes under six different treatments in the La Mancha freshwater wetland. **Table S3.** Repeated measures ANOVA table for Species richness (*S*), Diversity (*H*), and Equitability (*E*) under different disturbance treatments on five sampling events. **Table S4.** Repeated measures ANOVA table for Interstitial pH, electric conductivity, soil moisture, water level and soil Eh under different disturbance treatments on five sampling events in the wetland of La Mancha, Veracruz.
