# Supplementary material for: Shade treatment affects structure and recovery of invasive C4 African grass Echinochloa pyramidalis
Source: Ecol Evol. 2015 Mar 1;5(6):1327–42. doi: 10.1002/ece3.1434 (PMC4377275; doi:10.1002/ece3.1434)
Supplement: Supplementary file 1 [file ece30005-1327-sd1.docx]

**Appendix A. Supporting material**

ANOVA tables for cov*I*/cov*N,* percent cover of five dominant hydrophytes; biom*I*/biom*N*, aerial biomass of five dominant hydrophytes; diversity indicators and physicochemical characteristics mentioned in the Results section.

Table A1. Repeated measures ANOVA table for the ratio response for cov*I*/cov*N* and the percent cover response of five dominant hydrophytes over time in six treatments.

|  | Source of Variation | df | MS | *F* | *P* |
| --- | --- | --- | --- | --- | --- |
| cov*I*/cov*N* | |  |  |  |  |
|  | Blocks | 6 | 0.0000104 |  |  |
|  | Treatments | 5 | 0.00664 | 65.100 | <0.001 |
|  | Treatments x Blocks | 30 | 0.000102 |  |  |
|  | Time | 4 | 0.000308 | 14.110 | <0.001 |
|  | Time x Blocks | 24 | 0.0000218 |  |  |
|  | Treatments x Time | 20 | 0.000296 | 16.155 | <0.001 |
|  | Error (Treatments x Time) | 120 | 0.0000183 |  |  |
|  |  |  |  |  |  |
| *Echinochloa pyramidalis* | |  |  |  |  |
|  | Blocks | 6 | 0.0160 |  |  |
|  | Treatments | 5 | 9.607 | 81.231 | <0.001 |
|  | Treatments x Blocks | 30 | 0.118 |  |  |
|  | Time | 4 | 0.0782 | 5.385 | 0.003 |
|  | Time x Blocks | 24 | 0.0145 |  |  |
|  | Treatments x Time | 20 | 0.448 | 27.808 | <0.001 |
|  | Error (Treatments x Time) | 120 | 0.0161 |  |  |
|  |  |  |  |  |  |
| *Sagittaria lancifolia* | |  |  |  |  |
|  | Blocks | 6 | 0.0200 |  |  |
|  | Treatments | 5 | 0.346 | 16.445 | <0.001 |
|  | Treatments x Blocks | 30 | 0.0210 |  |  |
|  | Time | 4 | 0.318 | 37.984 | <0.001 |
|  | Time x Blocks | 24 | 0.00838 |  |  |
|  | Treatments x Time | 20 | 0.0368 | 4.934 | <0.001 |
|  | Error (Treatments x Time) | 120 | 0.00746 |  |  |
|  |  |  |  |  |  |
| *Pontederia sagittata* | |  |  |  |  |
|  | Blocks | 6 | 0.162 |  |  |
|  | Treatments | 5 | 4.575 | 27.063 | <0.001 |
|  | Treatments x Blocks | 30 | 0.169 |  |  |
|  | Time | 4 | 0.0415 | 3.757 | 0.016 |
|  | Time x Blocks | 24 | 0.0110 |  |  |
|  | Treatments x Time | 20 | 0.0110 | 2.179 | 0.005 |
|  | Error (Treatments x Time) | 120 | 0.00858 |  |  |
|  |  |  |  |  |  |
| *Fuirena simplex* | |  |  |  |  |
|  | Blocks | 6 | 0.105 |  |  |
|  | Treatments | 5 | 3.741 | 57.481 | <0.001 |
|  | Treatments x Blocks | 30 | 0.0651 |  |  |
|  | Time | 4 | 0.436 | 20.983 | <0.001 |
|  | Time x Blocks | 24 | 0.0208 |  |  |
|  | Treatments x Time | 20 | 0.135 | 5.421 | <0.001 |
|  | Error (Treatments x Time) | 120 | 0.0250 |  |  |
|  |  |  |  |  |  |
| *Hydrocotylle umbellata* | |  |  |  |  |
|  | Blocks | 6 | 0.0583 |  |  |
|  | Treatments | 5 | 0.142 | 4.843 | 0.002 |
|  | Treatments x Blocks | 30 | 0.0294 |  |  |
|  | Time | 4 | 0.380 | 29.342 | <0.001 |
|  | Time x Blocks | 24 | 0.0129 |  |  |
|  | Treatments x Time | 20 | 0.0679 | 4.208 | <0.001 |
|  | Error (Treatments x Time) | 120 | 0.0161 |  |  |

Table A2. Two-way ANOVA table for the ratio response for biom*I*/biom*N* and aerial biomass for five dominant hydrophytes under six different treatments in the La Mancha freshwater wetland.

|  | Source of Variation | df | MS | *F* | *P* |
| --- | --- | --- | --- | --- | --- |
| biom*I*/biom*N* | |  |  |  |  |
|  | Blocks | 6 | 0.00247 | 0.353 | 0.903 |
|  | Treatments | 5 | 0.195 | 27.860 | <0.001 |
|  | Error (Treatments) | 30 | 0.00701 |  |  |
|  |  |  |  |  |  |
| *Echinochloa pyramidalis* | |  |  |  |  |
|  | Blocks | 6 | 58059.208 | 0.257 | 0.953 |
|  | Treatments | 5 | 6273085.052 | 27.57 | <0.001 |
|  | Error (Treatments) | 30 | 225996.437 |  |  |
|  |  |  |  |  |  |
| *Sagittaria lancifolia* | |  |  |  |  |
|  | Blocks | 6 | 5129.800 | 1.288 | 0.293 |
|  | Treatments | 5 | 29496.233 | 7.405 | <0.001 |
|  | Error (Treatments) | 30 | 3983.317 |  |  |
|  |  |  |  |  |  |
| *Pontederia sagittata* | |  |  |  |  |
|  | Blocks | 6 | 7235.742 | 1.060 | 0.408 |
|  | Treatments | 5 | 73020.507 | 10.693 | <0.001 |
|  | Error (Treatments) | 30 | 6828.509 |  |  |
|  |  |  |  |  |  |
| *Fuirena simplex* | |  |  |  |  |
|  | Blocks | 6 | 25208.047 | 1.746 | 0.144 |
|  | Treatments | 5 | 484786.032 | 33.587 | <0.001 |
|  | Error (Treatments) | 30 | 14433.618 |  |  |
|  |  |  |  |  |  |
| *Hydrocotylle umbellata* | |  |  |  |  |
|  | Blocks | 6 | 146.365 | 1.120 | 0.375 |
|  | Treatments | 5 | 227.022 | 1.737 | 0.157 |
|  | Error (Treatments) | 30 | 130.734 |  |  |

Table A3. Repeated measures ANOVA table for Species richness (*S*), Diversity (*H*), and Equitability (*E*) under different disturbance treatments on five sampling events.

|  | Source of Variation | df | MS | *F* | *P* |
| --- | --- | --- | --- | --- | --- |
| Species richness | |  |  |  |  |
|  | Blocks | 6 | 0.129 |  |  |
|  | Treatments | 5 | 2.887 | 14.588 | <0.001 |
|  | Treatments x Blocks | 30 | 0.198 |  |  |
|  | Time | 4 | 0.462 | 8.024 | <0.001 |
|  | Time x Blocks | 24 | 0.0576 |  |  |
|  | Treatments x Time | 20 | 0.248 | 5.218 | <0.001 |
|  | Error (Treatments x Time) | 120 | 0.0476 |  |  |
|  |  |  |  |  |  |
| Diversity | |  |  |  |  |
|  | Blocks | 6 | 0.134 |  |  |
|  | Treatments | 5 | 2.592 | 15.047 | <0.001 |
|  | Treatments x Blocks | 30 | 0.172 |  |  |
|  | Time | 4 | 0.896 | 29.436 | <0.001 |
|  | Time x Blocks | 24 | 0.0305 |  |  |
|  | Treatments x Time | 20 | 0.209 | 5.562 | <0.001 |
|  | Error (Treatments x Time) | 120 | 0.0375 |  |  |
|  |  |  |  |  |  |
| Equitability | |  |  |  |  |
|  | Blocks | 6 | 0.0275 |  |  |
|  | Treatments | 5 | 0.158 | 10.810 | <0.001 |
|  | Treatments x Blocks | 30 | 0.0146 |  |  |
|  | Time | 4 | 0.169 | 16.415 | <0.001 |
|  | Time x Blocks | 24 | 0.0103 |  |  |
|  | Treatments x Time | 20 | 0.0546 | 4.580 | <0.001 |
|  | Error (Treatments x Time) | 120 | 0.0119 |  |  |

Table A4. Repeated measures ANOVA table for Interstitial pH, electric conductivity, soil moisture, water level and soil Eh under different disturbance treatments on five sampling events in the wetland of La Mancha, Veracruz.

|  | Source of Variation | df | MS | *F* | *P* |
| --- | --- | --- | --- | --- | --- |
| pH | |  |  |  |  |
|  | Blocks | 6 | 0.168 |  |  |
|  | Treatments | 5 | 0.0149 | 0.691 | 0.634 |
|  | Treatments x Blocks | 30 | 0.0215 |  |  |
|  | Time | 4 | 9.860 | 76.653 | <0.001 |
|  | Time x Blocks | 24 | 0.129 |  |  |
|  | Treatments x Time | 20 | 0.0192 | 1.265 | 0.216 |
|  | Error (Treatments x Time) | 120 | 0.0152 |  |  |
|  |  |  |  |  |  |
| Electric conductivity | |  |  |  |  |
|  | Blocks | 6 | 0.00823 |  |  |
|  | Treatments | 5 | 0.00643 | 1.656 | 0.176 |
|  | Treatments x Blocks | 30 | 0.00388 |  |  |
|  | Time | 4 | 0.0303 | 5.370 | 0.003 |
|  | Time x Blocks | 24 | 0.00565 |  |  |
|  | Treatments x Time | 20 | 0.00366 | 1.454 | 0.111 |
|  | Error (Treatments x Time) | 120 | 0.00252 |  |  |
|  |  |  |  |  |  |
| Soil moisture | |  |  |  |  |
|  | Blocks | 6 | 0.00523 |  |  |
|  | Treatments | 5 | 0.00203 | 0.911 | 0.487 |
|  | Treatments x Blocks | 30 | 0.00223 |  |  |
|  | Time | 4 | 0.0374 | 17.368 | <0.001 |
|  | Time x Blocks | 24 | 0.00215 |  |  |
|  | Treatments x Time | 20 | 0.00157 | 0.961 | 0.513 |
|  | Error (Treatments x Time) | 120 | 0.00164 |  |  |
|  |  |  |  |  |  |
| Water level | |  |  |  |  |
|  | Blocks | 6 | 3.648 |  |  |
|  | Treatments | 5 | 14.503 | 5.581 | <0.001 |
|  | Treatments x Blocks | 30 | 2.598 |  |  |
|  | Time | 4 | 749.536 | 141.284 | <0.001 |
|  | Time x Blocks | 24 | 5.305 |  |  |
|  | Treatments x Time | 20 | 5.364 | 2.507 | 0.001 |
|  | Error (Treatments x Time) | 120 | 2.140 |  |  |
|  |  |  |  |  |  |
| Soil Eh | |  |  |  |  |
|  | Blocks | 6 | 1598.563 |  |  |
|  | Treatments | 5 | 592.781 | 1.967 | 0.112 |
|  | Treatments x Blocks | 30 | 301.311 |  |  |
|  | Time | 4 | 520581.748 | 462.460 | <0.001 |
|  | Time x Blocks | 24 | 1125.680 |  |  |
|  | Treatments x Time | 20 | 415.991 | 1.802 | 0.028 |
|  | Error (Treatments x Time) | 120 | 230.789 |  |  |
